# Up-regulation of macrophage wnt gene expression in adenoma-carcinoma progression of human colorectal cancer

**DOI:** 10.1038/sj.bjc.6690721

**Published:** 1999-10

**Authors:** K Smith, T D Bui, R Poulsom, L Kaklamanis, G Williams, A L Harris

**Affiliations:** ICRF Molecular Oncology Laboratory, John Radcliffe Hospital, Oxford, OX3 9DS, UK; Wellcome Trust, Nuffield Orthopaedic Hospital, Oxford, UK; Histopathology Unit, ICRF, 44 Lincoln's Inn Fields, London, WC2A 3PX, UK; Pathology Department, Onassis Cardiac Surgery Center, Sigrov Av 356, Athens, 17674, Greece

**Keywords:** human colorectal cancer, in situ hybridization, macrophages, tumour progression, wnt genes

## Abstract

Defects in the APC-β-catenin pathway are common in colon cancer. We investigated whether aberrant regulation of upstream ligands stimulating this pathway occur in colon cancer. Using RNAase protection analysis, six out of eight wnt genes were expressed in 14 matched cases of normal, adenomatous and malignant colorectal tissues. Wnt 2 and wnt 5a were significantly up-regulated in the progression from normal through adenoma to carcinoma. Transcripts for wnts 4, 7b, 10b and 13, but not wnt 2 and wnt 5a were detected in several colorectal cell lines. In situ hybridization demonstrated that wnt 2 and wnt 5a transcripts were mainly in the lamina propria/stroma region with labelling predominantly in macrophages. Immunostaining with CD68 confirmed the wnt-expressing cells as macrophages. These results show a major difference in wnt expression in colon cancer compared to colon adenomas and suggest stromal wnt expression may play a role in tumour progression. © 1999 Cancer Research Campaign


*British Journal of Cancer* (1999) **81**(3), 496–502
© 1999 Cancer Research Campaign
Article no. bjoc.1999.0721

### Up-regulation of macrophage wnt gene expression in adenoma-carcinoma progression of human colorectal cancer

**K Smith[1], TD Bui[2], R Poulsom[3], L Kaklamanis[4], G Williams[3] and AL Harris[1]**

[1]ICRF Molecular Oncology Laboratory, John Radcliffe Hospital, Oxford OX3 9DS, UK; [2]Wellcome Trust, Nuffield Orthopaedic Hospital, Oxford, UK; [3]Histopathology Unit, ICRF, 44 Lincoln's Inn Fields, London WC2A 3PX, UK; [4]Pathology Department, Onassis Cardiac Surgery Center, Sigrov Av 356, Athens, 17674, Greece

**Summary** Defects in the APC-β-catenin pathway are common in colon cancer. We investigated whether aberrant regulation of upstream ligands stimulating this pathway occur in colon cancer. Using RNAase protection analysis, six out of eight wnt genes were expressed in 14 matched cases of normal, adenomatous and malignant colorectal tissues. Wnt 2 and wnt 5a were significantly up-regulated in the progression from normal through adenoma to carcinoma. Transcripts for wnts 4, 7b, 10b and 13, but not wnt 2 and wnt 5a were detected in several colorectal cell lines. In situ hybridization demonstrated that wnt 2 and wnt 5a transcripts were mainly in the lamina propria/stroma region with labelling predominantly in macrophages. Immunostaining with CD68 confirmed the wnt-expressing cells as macrophages. These results show a major difference in wnt expression in colon cancer compared to colon adenomas and suggest stromal wnt expression may play a role in tumour progression. © 1999 Cancer Research Campaign

**Keywords:** human colorectal cancer; in situ hybridization; macrophages; tumour progression; wnt genes

Human tumorigenesis is associated with the accumulation of mutations in oncogenes and in tumour suppressor genes (Fearon and Vogelstein, 1990). Mutations in the adenomatous polyposis coli (APC) tumour suppressor gene occur in the germline of patients with familial adenomatous polyposis (FAP), an inherited disease predisposing to colorectal cancers (Groden et al, 1991; Miyoshi et al, 1992). The majority of colorectal cancers are, however, sporadic, occurring in patients without FAP and in the majority of such cases mutations occur (Powell et al, 1992). Almost all APC mutations disrupt the coding sequence in the 3′ half of APC (Miyoshi et al, 1992; Powell et al, 1992) producing truncated APC proteins (Smith et al, 1993), implying that truncated APC proteins may promote tumour cell growth.

Both wild-type and mutant APC proteins (Su et al, 1993; Rubinfield et al, 1995) interact with the E-cadherin associated proteins α- and β-catenin. However only wild-type APC is able to down-regulate β-catenin by enhancing degradation when expressed exogenously in colon cancer cells (Munemitsu et al, 1995). This activity is localized between amino acid residues 1020–1169 (Su et al, 1993), which are absent in mutant APC, implying that this region is essential for the down-regulation of β-catenin and regulation of cell growth.

In mammalian systems, transfection of wnt 1 into PC12, AtT20 and C57MG cells leads to an accumulation of catenins (Hinck et al, 1994), and recently Papkoff et al (Papkoff et al, 1996) have shown that in C57MG cells exogenous wnt 1 results in accumulation of monomeric catenins together with stabilization of the APC–catenin complex. Antagonists also regulate this pathway. Exogenous expression of axin, the gene product of the mouse fused gene (Zeng et al, 1997) results in a reduction in β-catenin levels in SW 480 colon cancer cells (Hart et al, 1998; Nakamura et al, 1998). Furthermore, Hart et al (1998) showed that axin forms a tetrameric complex with glycogen synthase kinase 3β (GSK3β), β-catenin and APC resulting in increased phosphorylation of both APC and β-catenin and ultimately leading to increased β-catenin degradation. The region of APC necessary for binding to axin is deleted in the truncated APC mutants found in colon cancer (Hart et al, 1998).

Activation of the wnt-signalling pathway inhibits GSK3β activity resulting in stabilization of monomeric β-catenin pools (Papkoff et al, 1996), which are then able to associate with T-cell factor (Tcf) transcription factor to enhance transcription of, as yet, unknown genes (Korinek et al, 1997). Pennica et al (1999) have shown that, in C57MG cells transfected with wnt 1, there is up-regulation of two genes, WISP-1 and Wisp-2, and furthermore in 84% of a series of human colorectal tumours WISP-1 mRNA was overexpressed when compared with patient-matched normal mucosa. This wnt stabilization of β-catenin pools is analogous to the situations produced either by truncations in the APC gene product (Munemitsu et al, 1995) or by mutations in serine/threonine phosphorylation sites in β-catenin (Morin et al, 1997). Mutations in APC are, however, present in adenomas and therefore further changes are required to progress to carcinoma. A second APC gene, named APCL or APC2, has recently been cloned (Nakagawa et al, 1998; van Es et al, 1999) and shown to be involved in controlling β-catenin–Tcf-mediated transcription (van Es, 1999).

Studies in the nematode, *Caenorhabditis elegans* (Rocheleau et al, 1997), have led to the identification of a set of maternally

*Received 14 December 1998*
*Revised 9 April 1999*
*Accepted 22 April 1999*

*Correspondence to:* K Smith expressed genes, which are related to genes of the wnt-mediated signalling pathway. They showed that mutations in these novel genes disrupted normal embryonic endoderm induction resulting in an abnormal gut phenotype. In *Drosophila* posterior midgut morphogenesis the diffusible polypeptides, wnt 1 and decapentaplegic (Dpp, a member of the transforming growth factor β (TGF-β) family) modulate activation of genes under control of the HOX gene, abdomenal A (abd A) (Bilder et al, 1998). If a wnt-like pathway is important in normal gut development subsequent alterations in this pathway may be involved in colonic tumour progression.

We previously showed that wnts 2, 4, 5a and 7b are up-regulated in diseased states of human breast tissue (Huguet et al, 1994; LeJeune et al, 1995). In the present study we have analysed matched normal, adenomatous and malignant tissues taken from each of 14 patients with colon cancer. The expression of eight wnt genes have been quantified by RNAase protection analysis and genes differentially up-regulated localized by in-situ mRNA analysis. Additionally a range of colon adenoma and carcinoma cell lines were analysed to assess wnt expression in vitro models of human colon cancer.

### MATERIALS AND METHODS

### Tissue samples

Matched normal, adenomatous and malignant tissues were each resected from 14 patients at the John Radcliffe Hospital, Oxford. Normal samples were removed from sites greater than 2 cm distant from the carcinoma, and adenoma samples were removed from sites greater than 1 cm from the carcinoma. All samples were carried to the laboratory on ice whereupon they were examined by a pathologist, snap-frozen and stored at –196°C until used. The samples were from independent lesions and assessed pathologically to ensure that they were distinct adenomas or carcinomas.

### RNA preparation

Ribonucleic acid was extracted from tissues using the acid guanidinium thiocyanate phenol chloroform method (Chomczynski et al, 1987). Aliquots (2 µg) of RNA were electrophoresed through 1% agarose gels and only those preparations with a 28S:18S ribosomal ratio of approximately 2 were used for further analysis.

### RNAase protection analysis

In all assays, 10 µg of total RNA were hybridized overnight at 55°C with a cocktail containing: $10^5$ cpm of [α-$^{32}$P]CTP-labelled wnt antisense riboprobe, $5 \times 10^4$ cpm of [α-$^{32}$P]CTP-labelled glyceraldehyde-3-phosphate dehydrogenase (GAPDH) antisense riboprobe, $2 \times 10^3$ cpm of [α-$^{32}$P]CTP-labelled GAPDH sense riboprobe and 20 µg carrier tRNA.

In quantifying ribonuclease protection data GAPDH is commonly used as an internal control to standardize for losses during the experiment. However, GAPDH is not a reliable internal control when assaying mRNA expression in tumours. A more accurate method of standardization is to include [α$^{32}$P]CTP-labelled sense and antisense GAPDH in the ratio 1:20 (counts per minute) in the hybridization cocktail (Scott et al, 1997). Quantitation of the exogenous GAPDH sense–antisense mRNA duplex provides a more accurate determination of losses during the procedure in addition to providing a measure of the endogenous GAPDH, which is also quantitatively detected on the gel.

In order to ensure that equal amounts of tissue RNA were taken for hybridization, total RNA samples were measured spectrophotometrically and the values obtained were then confirmed by analysing 2-µg aliquots by agarose gel electrophoresis and visualization of the 18S and 28S ribosomal RNAs.

Protected fragments were separated on 6% acrylamide/urea sequence gels and subsequently exposed to X-ray film at –80°C with intensifying screens. Exposure times varied between 1 h (GAPDH signal) and 14 days (low abundance wnt signals). Signals were quantified using either a Bioimage analyser (Millipore) or a Phosphorimager 425 (Molecular Dynamics).

### Construction of RNAase protection templates

Riboprobe templates for wnt 2 and wnt 3 were generated by PCR amplification as described previously (Huguet et al, 1994) to give fragments of 406 and 424 bp respectively. Wnt 4 and Wnt 7b riboprobe templates were isolated according to published procedures (Gavin et al, 1990) and described in more detail (Huguet et al, 1994); fragment sizes were 360 and 372 bp respectively. A probe template for wnt 11 was constructed by RT-PCR amplification of human breast cancer RNA to give a fragment of 351 bp. A wnt 5a riboprobe template was generated as described (LeJeune et al, 1995), giving a fragment of 384 bp and a wnt 10b probe template was generated by PCR amplification resulting in a fragment of 370 bp (Bui et al, 1997*b*). The degree of homology between wnt riboprobes was sufficiently low enough to exclude any possibility of each probe cross-hybridizing with other wnt family messenger RNAs. The highly stringent conditions of the RNAase protection assay, in conjunction with riboprobe design, also ensured no cross-interference during analysis. A 120 bp fragment of human GAPDH was cloned into plasmid pBluescript SK$^+$. Antisense [α-$^{32}$P]CTP-labelled riboprobes were synthesized from their linearized respective templates using the appropriate RNA polymerase. In the case of GAPDH sense [α-$^{32}$P]CTP-labelled riboprobe was also synthesized.

### Isolation of wnt 13 cDNA by RT-PCR

Heat denatured breast tumour RNA was reverse transcribed for 1 h at 37°C in a 50 µl reaction volume containing 1 µg degenerate reverse transcription (RT) oligonucleotide (5′-GCA$^{G}/_{A}$CACCA$^{G}/_{A}$TG$^{G}/_{A}{}^{A}/_{T}$A-3′), corresponding to amino acid wnt consensus region FHWCC0 and 2.5 units murine reverse transcriptase. Aliquots of reverse transcribed RNA and negative controls were used in 50 µl polymerase chain reaction (PCR) reactions containing 1 µg each of forward and reverse degenerate PCR oligonucleotides (forward oligonucleotide corresponding to amino acid wnt consensus region CKCHG; 5′-TG$^{T}/_{C}$AA$^{A}/_{G}$TG$^{T}/_{C}$C-A$^{C}/_{T}$GG$^{G}$/A/$_{T}$-3′, reverse oligonucleotide corresponding to amino acid wnt consensus region CCGRG; 5′-$^{G}/_{C}$CC$^{A}$/C/T/$_{G}$C$^{T}/_{G}{}^{A}$/C/$_{G}$C-C$^{G}/_{A}$CAGCA-3′). Five microlitres of the PCR reactions were electrophoresed in a 2% agarose gel and DNA visualized by ethidium bromide staining. Bands were cut out of the gel, subcloned into the pCR®2 vector and sequenced by the dideoxynucleotide chain termination method using a sequenase$^{R}$ kit. The sequence obtained by us matches that recently published (Katoh et al, 1996).

© 1999 Cancer Research Campaign 

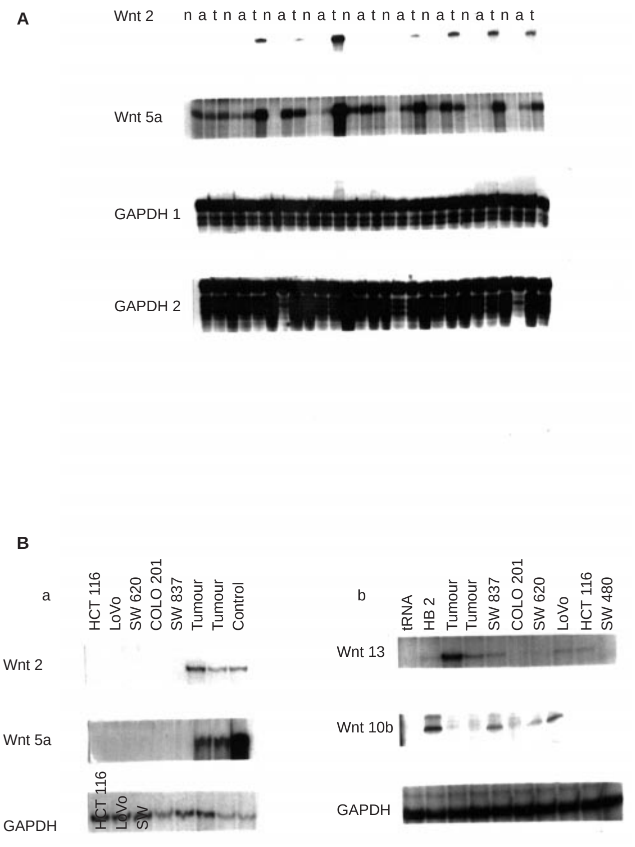

**Figure 1** Ribonuclease protection analysis showing wnt 2 gene expression in colorectal tissues and cell lines. (**A**) wnt 2 and wnt 5a mRNA levels together with corresponding GAPDH sense – antisense mRNA hybrid levels (GAPDH 1) as loading control in nine matched normal, adenoma and tumour specimens. The endogenous GAPDH mRNA levels for each sample are also show (GAPDH 2). n = normal, a = adenoma and t = tumour RNAs respectively. (**B**) wnt2 and wnt 5a mRNA levels (a), wnt 13 and wnt 10b mRNA levels (b). GAPDH sense-antisense hybrid levels are shown as loading controls. Tumour and HB 2 breast cell RNA samples were included as positive controls. tRNA = transfer RNA was included as negative control.

### Cell lines and culture conditions

The following human colorectal adenocarcinoma cell lines were used in this study: LOVO, SW620, SW837, SW480, COLO201 and HT29. The line HCT116 is a human colorectal carcinoma cell. The lines AA/C1, RG/C2 and BH/C1 are adenoma cell lines, whilst AA/C1/SB/10C is a chemically transformed tumorigenic variant of the AA/C1 line. Breast HB2 cells were used as positive control.

Cells were grown in Dulbecco's modified Eagle's medium containing 10% fetal bovine serum in a humidified incubator under 95% air, 5% carbon dioxide. RNA was extracted from cells when approximately 50–70% confluent.

### In situ hybridization

In situ hybridization was carried out essentially as described by Senior et al (1988). Briefly, 4-µm sections were cut from formalin-fixed, paraffin-embedded blocks on TESPA (7,3-aminopropyl-triethoxy silane)-treated slides and dried overnight at 37°C. Sections were permeabilized with proteinase-K, fixed in 4% paraformaldehyde, air dried and hybridized overnight at 55°C with $10^6$ cpm of [$^{35}$S]UTP-labelled antisense riboprobes for wnt 2 and wnt 5a in 10 µl hybridization buffer. The slides were washed several times in 50% formamide–4 × standard saline citrate (SSC) at 55°C to remove unhybridized probe, followed by RNAase treatment to digest single-stranded and imperfectly hybridized domains and washing extensively to a final stringency of 0.5 × SSC at 65°C for 30 min. Slides were dipped in Ilford K5 emulsion and autoradiographed at 4°C for 7 or 14 days. Latent images were developed with Kodak D-19 and counterstained with Giemsa. As a positive control for the presence of mRNA, sections were hybridized with a β-actin antisense [$^{35}$S]UTP-labelled riboprobe.

### Immunochemical analysis of CD68 protein

Deparaffinized, rehydrated tissue sections were treated with 3% hydrogen peroxide to quench endogenous peroxidase activity. Sections were microwaved 4 min in citrate buffer, rinsed with distilled water and placed in TBS for 5 min. Mouse monoclonal anti-CD68 antibody, KP1 (Pulford et al, 1989) (Dako, Glostrup, Denmark) in TBS–1% bovine serum albumin (BSA) was added and incubated 30 min at room temperature. After washing in TBS, streptABComplex/HRP conjugated to goat anti-mouse antibody was added for 30 min room temperature. Sections were again washed with TBS prior to development with diaminobenzidine as chromagen. Slides were finally washed in distilled water, counterstained with haematoxylin and mounted in Apathy's aqueous medium.

### Statistical analysis

Expression values of each wnt gene in matched normal, adenoma and carcinoma samples from the same patient were compared using Wilcoxon matched pairs sign test. The Mann–Whitney *U*-test was used to compare expression values for each wnt gene in normal, adenoma and carcinoma samples.

### RESULTS

### Patient characteristics

Normal, adenomatous and carcinomatous tissues were removed from each of 14 patients with colorectal cancer. The mean and median ages were both 69 years (range 54–82 years). Regional nodes were sampled in all patients at the time of surgery. Eight patients had involvement of lymph nodes. Three cancers were rectal, two were caecal and nine were colonic. Three tumours were well- and 11 were moderately differentiated. One tumour was confirmed as Dukes' stage A, 5 as Dukes' stage B and 8 as Dukes' stage C.

### Sequence analysis of gene fragments amplified by degenerate RT-PCR

Purification, cloning and sequencing of the fragments isolated after RT-PCR resulted in the isolation of a novel wnt gene (wnt 13) whose sequence together with its corresponding amino acid sequence is in agreement with that recently reported (Katoh et al, 1996) (results not shown).

 © 1999 Cancer Research Campaign

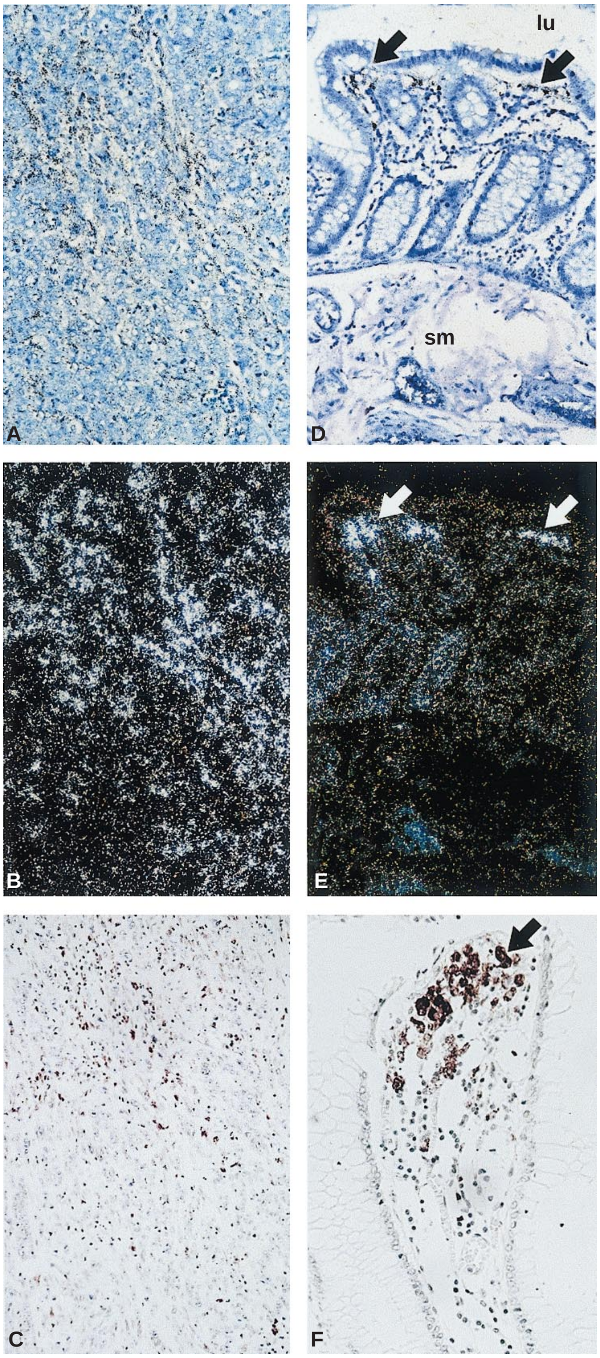

**Figure 2** In situ RNA hybridization of wnt 5a and anti-CD68 immunostaining in human colorectal tissues. (**A**) Poorly differentiated carcinoma of large bowel, light field. (**B**) Poorly differentiated carcinoma of large bowel, dark field showing stromal areas with up-regulated wnt 5a expression. (**C**) Poorly differentiated carcinoma of large bowel showing anti-CD68 immunostaining. (**D**) Normal large bowel, light field. (**E**) Normal large bowel, dark field. (**F**) Normal large bowel showing anti-CD68 immunostaining. Arrows indicate the lamina propria containing wnt 5a-expressing macrophages; Lu=lumen; SM=submucosa. All exposures were taken at ×285 magnification

### Expression of wnt genes in human colorectal tissues

Figure 1A shows a representative RNAase protection analyses of wnt 2 and wnt 5a in colorectal tissues. The results for all wnt expression profiles are tabulated in Table 1. The median wnt2 mRNA expression in tumours was up-regulated 28-fold

**Table 1** Wnt gene expression in matched normal, adenoma and carcinoma specimens. Messenger RNA transcripts were assayed by RNase protection assay. The values for the mean ± SD, median and range, in densitometric units, and number of specimens are shown.

| Wnt mRNA | | Normal | Adenoma | Carcinoma |
|---|---|---|---|---|
| Wnt 2 | median | 2.3 (n = 14) | 3.4 (n = 14) | 64.5 (n = 14) |
| | range | 0–4.7 | 0–26.2 | 2.0–1291.0 |
| | mean ± SD | 1.83 ± 1.69 | 5.03 ± 6.73 | 168.7 ± 321.1 |
| Wnt 3 | median | 0 (n = 9) | 0(n = 9) | 0.3 (n = 9) |
| | range | 0–0.1 | 0–0.5 | 0–3.5 |
| | mean ± SD | 0.01 ± 0.02 | 0.06 ± 0.16 | 0.72 ± 1.12 |
| Wnt 4 | median | 1.8 (n = 12) | 5.8 (n = 13) | 8.8 (n = 14) |
| | range | 0–27.1 | 0–22.6 | 0–51.8 |
| | mean ± SD | 5.1 ± 8.0 | 6.4 ± 5.6 | 16.5 ± 17.2 |
| Wnt 5a | median | 11.7 (n = 14) | 26.2 (n = 14) | 49.3 (n = 14) |
| | range | 1.1–50.6 | 0–100.2 | 5.9–178.5 |
| | mean ± SD | 15.4 ± 12.5 | 32.7 ± 31.5 | 62.2 ± 48.0 |
| Wnt 7a | median | N.D. (n = 5) | N.D. (n = 5) | N.D. (n = 5) |
| | range | | | |
| | mean ± SD | | | |
| Wnt 10b | median | N.D. (n = 5) | N.D. (n = 5) | N.D. (n = 5) |
| | range | | | |
| | mean ± SD | | | |
| Wnt 11 | median | N.D. (n = 5) | N.D. (n = 5) | N.D. (n = 5) |
| | range | | | |
| | mean ± SD | | | |
| Wnt 13 | median | 0 (n = 5) | 0 (n = 5) | 0 (n = 5) |
| | range | 0–3.6 | 0–0.2 | 0–1.7 |
| | mean ± SD | 1.17 ± 1.38 | 0.03 ± 0.06 | 0.50 ± 0.66 |

N>D> = Not detected.

($P$ = 0.0001, Wilcoxon test) compared with normal and 19-fold ($P$ = 0.0002, Wilcoxon test) compared with adenomatous tissues.

In relation to the other wnt mRNAs analysed the message for wnt 5a was most abundantly expressed. The carcinomas also had higher levels of this message compared with normals ($P$ = 0.0018, Wilcoxon test) and with adenomas ($P$ = 0.0129, Wilcoxon test). Levels of wnt 5a were comparable to those observed in human breast cancers (LeJeune et al, 1995). When the above data for wnt 2 and wnt 5a were re-analysed using the Mann–Whitney $U$-test the differences remained statistically significant with very similar $P$-values (data not shown).

Wnt 13 showed no differential regulation between carcinoma and non-carcinoma samples, and the message levels for wnt 11 and wnt 10b were too low to be detected by RNAase protection analysis. Wnt 7b expression was detected in one tumour sample only (Table 1).

### Expression of wnt genes in human colorectal cell lines

Figure 1B shows RNA protection analysis of wnt 2 and wnt 5a (a) and of wnt 10b and wnt 13 (b). Table 2 depicts all wnt expression profiles in 11 colorectal and one breast (HB2) cell lines. The abundance of all wnt mRNAs was considerably less than in tissues, and furthermore those wnt genes expressed in tissues were absent in cell lines whilst wnt genes not expressed in tissues were expressed in cells. Hence wnt 2, and to a lesser degree wnt 5a, which were up-regulated in carcinomas compared to normal tissue were totally absent in all colon cell lines analysed. Wnt 7b and wnt 10b, which were absent (with one exception) in colorectal tissues, were present, albeit at very low abundance in several carcinoma cell lines. Wnt 13 mRNA was present in three colon carcinoma cell

*© 1999 Cancer Research Campaign* 

**Table 2** Wnt gene expression in colorectal adenoma and carcinoma cell lines (for details see methods section) and in one breast cell (HB 2). Messenger RNA transcripts were measured by RNase protection assay. Levels were graded – (negative) through to +++ (strongly positive).

| Cell line | Wnt2 | Wnt4 | Wnt 5a | Wnt 7b | Wnt 10b | Wnt 13 |
|---|---|---|---|---|---|---|
| HCT 116 | – | +/– | – | +/– | – | + |
| LOVO | – | +/– | – | + | + | + |
| SW 620 | – | +/– | – | +/– | +/– | – |
| SW 837 | – | +/– | – | +/– | +/– | + |
| COLO 201 | – | – | – | +/– | – | – |
| SW 480 | – | +/– | +/– | nd | – | – |
| HT 29 | – | nd | – | – | – | nd |
| AA/C1 | – | nd | – | – | – | nd |
| AA/C1/SB/10C | – | nd | – | – | – | nd |
| RG/C2 | – | nd | – | – | – | nd |
| BH/C1 | – | nd | – | – | – | nd |
| HB 2 | – | nd | +++ | ++ | + | nd |

nd denotes not determined.

lines as well as in tissues. No wnt gene expression was detected in any of the adenoma cell lines (even after 14 days exposure).

### In situ hybridization

Transcripts for both wnt 2 and wnt 5a were detected in several colorectal tissues. The general level of expression of wnt 5a was higher than that for wnt 2, which is in keeping with the RNAase protection data where wnt 5a was clearly the most abundantly expressed message. Background levels were higher for wnt 2. Wnt 5a labelling was predominantly present in the stroma/lamina propria in normal, adenoma and carcinoma tissues. Representative light (Figure 2A) and dark (Figure 2B) field exposures of a poorly differentiated carcinoma of the large bowel show high expression of wnt 5a in cells within stromal elements, and this was especially clear near invasion fronts and ulcerated regions. Some of the positively staining cells appeared to be macrophages, whereas others looked like fibroblasts. Figure 2D and E show representative light and dark fields respectively of a section of normal large bowel with wnt 5a expression in the lamina propria beneath the surface epithelium. The only signals above background were in the stroma, principally in cells between the tops of crypts. The morphology of the positively labelled cells together with their location beneath the surface epithelium in normal tissues indicated them to be macrophages. Some positive cells appeared to be stromal fibroblasts. However, it is not uncommon for macrophages to assume a fibroblastic shape. The expression pattern for wnt 2 was similar to that seen for wnt 5a.

Immunostaining of the above sections with anti-CD 68 antibody (Figure 2C, large bowel poorly differentiated carcinoma; and Figure 2F, normal large bowel) identified the wnt-expressing cells to be macrophages.

### DISCUSSION

Wnt genes were initially isolated from mouse mammary carcinomas by insertional mutagenesis, but we have shown that they are also up-regulated in human hormone-dependent cancers of the breast (Huguet et al, 1994) and endometrium (Bui et al, 1997*a*). Wnts are members of a multigene family with over 14 genes in humans and most are conserved in *Xenopus*, suggesting specialized roles for each gene product. We therefore examined the potential role of wnts in progression from adenomas to carcinomas which could be via wnt-induced inactivation of GSK-3β. The presence of mutant APC gene products, which are unable to degrade β-catenin, would also result in the elevation of monomeric β-catenin levels. Support for this idea comes from a recent publication showing that transfection of wild-type APC gene product into colon carcinoma cells containing mutant APC removed β-catenin from Tcf-4 and abrogated the transcriptional transactivation (Korinek et al, 1997). Since in those colorectal cancers maintaining wild-type APC gene product, wnt signalling could further enhance β-catenin levels.

Our results showed that wnt 2 was up-regulated 28-fold in cancer samples. This contrasts with our studies of breast (Huguet et al, 1994) and endometrial (Bui et al, 1997*a*) cancer where wnts 5a, 7b and 10b, but not wnt 2, were up-regulated. It may be that several wnt genes are involved in the growth and development of individual organs and each cancer type may exhibit a deregulation in the expression of one or more wnt genes controlling normal development within that organ. In colorectal cancer the situation is further complicated, as discussed below, by the infiltration of macrophages expressing relatively high levels of selected wnt genes. While this work was in progress Vider et al (1996) showed that, using RT-PCR, wnt 2 was up-regulated in colon cancer compared with matching normal mucosa samples. In contrast to our results they found that in seven adenomas from separate patients wnt 2 expression was similar to that seen in carcinomas. We are unsure as to why this is other than that different methodologies (RT-PCR opposed to RNAase protection and non-matched samples compared to matched samples) were used. It is possible that had Vider et al (1996) assayed expression levels in matching carcinomas and adenomas the levels of wnt 2 may have been considerably higher in the carcinoma compared to the adenoma. Of interest is the observation that the range of wnt 2 expression was far greater in our study compared to theirs. In addition, they did not investigate the site of expression.

When adenoma and adenocarcinoma cell lines were studied for expression of those wnt genes most markedly up-regulated in colorectal carcinomas there was no detectable expression, in contrast to the situation in breast and endometrial cancer cell lines. This suggested that the up-regulation may be via a paracrine pathway. In situ hybridization clearly showed expression of wnt 2 and wnt 5a in the stroma/lamina propria of normal, adenomatous and carcinomatous colon samples. Immunohistochemical staining with monoclonal antibodies to CD68 revealed that the majority of positively labelled cells were macrophages, although some of the positive cells had a fibroblast morphology. Our failure to assay wnt 2 and wnt 5a expression in our panel of cell lines could be explained on the basis that in the tissue samples expression of these two genes was predominantly in infiltrating macrophages and not in tissue epitheliums. Although this is the first report of expression of wnt genes by macrophages or haemopoietic cells in adults, it has recently been shown that foetal murine haemopoietic tissues express wnts 1 and 5a (Austin et al, 1997). In addition, wnts 1, 5a and 10b directly stimulate the proliferation of murine liver haemopoietic stem/progenitor cell populations in culture, thereby functioning as haemopoietic regulatory factors. Wnt gene expression, overall, was very low in cell lines compared to carcinomas. Of those genes expression there was little variation between individual cell lines. This may imply that in these cell lines wnt gene function can be mediated by similar gene members.

 *© 1999 Cancer Research Campaign*

Increased microphage infiltration into colon tumours compared to normal mucosa was shown recently in a study involving expression of the angiogenic enzyme thymidine phosphorylase (Takebayashi et al, 1997). This enzyme was more highly expressed in cancers primarily due to the inflammatory infiltrate. It is possible that the presence of APC mutations in colorectal cancers, resulting in elevated β-catenin pools, could increase signalling, via β-catenin and cadherin, between malignant cells and infiltrating macrophages leading to increased wnt 2 transcription. A correlation between wnt 2 expression and APC status within colorectal carcinomas would therefore be of interest. However, the up-regulation of wnt 2 in inflammatory infiltrates is clearly not a general mechanism leading to an increased expression in cancer since we did not see it in endometrial cancers (Bui et al, 1997*a*). In breast cancer there was a shift in compartmental expression between epithelium and stroma, but no increase overall (Dale et al, 1996). Thus macrophages in different sites may have different activation or differentiation pathways.

It will be of interest to analyse stromal macrophage wnt 2 expression in co-culture with colon cells with and without APC mutations and since wnt proteins are heparin-binding growth factors they may be a target for therapy with heparin-like drugs. The demonstration of a role for macrophages in two different pathways of colonic tumour progression, i.e. angiogenesis and the wnt/APC/catenin pathway highlights their potential as a target for drug therapy.

### ACKNOWLEDGEMENTS

The authors would like to thank Drs M Pignatelli (London), C Paraskeva (Bristol) and A Rowan (Oxford) for generously supplying several cell lines used in this study. Financial support was provided by the Imperial Cancer Research Fund.

### REFERENCES

Austin TW, Solar GP, Ziegler FC, Liem L and Matthews W (1997) A role for the wnt gene family in hematopoiesis: expansion of multilineage progenitor cells. *Blood* **89**: 3624–3635

Bilder D, Graba Y and Scott MP (1998) Wnt and TGFβ signals subdivide the AbdA Hox domain during *Drosophila mesoderm* patterning. *Development* **125**: 1781–1790

Bui TD, Zhang L, Rees MCP, Bicknell R and Harris AL (1997*a*) Expression and hormone regulation of wnt 2, 3, 4, 5a, 7a, 7b and 10b in normal human endometrium and endometrial carcinoma. *Br J Cancer* **75**: 1131–1136

Bui T, Rankin J, Smith K, Huguet EL and Harris AL (1997*b*) Cloning and expression of human wnt 10b. *Oncogene* **11** 11–22

Chomczynski P and Sacchi N (1987) Single step method for RNA isolation by acid guanidinium thiocyanate-phenol-chloroform extraction. *Anal Biochem* **162**: 156–159

Dale TC, Weber Hall SJ, Smith K, Huguet EL, Jayatilake H, Gusterson BA, Shuttleworth G, O'Hare M and Harris AL (1996) Compartment switching of WNT-2 expression in human breast tumors. *Cancer Res* **56**: 4320–4323

Fearon ER and Vogelstein B (1990) A genetic model for colorectal tumorigenesis. *Cell* **61**: 759–767

Gavin BJ, McMahon JA and McMahon AP (1990) Expression of multiple novel wnt-1/int-1-related genes during fetal and adult mouse development. *Genes Dev* **4**: 2319–2332

Groden J, Thliveris A, Samowitz W, Carlson M, Gelbert L, Albertson H, Joslyn G, Stevens J, Spirio L, Robertson M, Sargeant L, Krapcho K, Wolff E, Burt R, Hunghes JP, Warrington J, McPherson J, Wasmuth J, LePaslier D, Abderrahim H, Cohen D, Leppert M and White R (1991) Identification and characterization of the familial adenomatous polyposis coli gene. *Cell* **66**: 589–600

Hart MJ, de los Santos R, Albert IN, Rubinfeld B and Polakis P (1998) Downregulation of beta catenin by human Axin and its association with the APC tumor suppressor, beta-catenin and GSK3 beta. *Curr Biol* **8**: 573–581

Hinck L, Nelson WJ and Papkoff J (1994) Wnt-1 modulates cell–cell adhesion in mammalian cells by stabilizing B-catenin binding to the cell adhesion protein cadherin. *J Cell Biol* **124**: 729–724

Huguet EL, McMahon JA, McMahon AP, Bicknell R and Harris AL (1994) Differential expression of human wnt genes 2, 3, 4 and 7b in human breast cell lines and normal and disease states of human breast tissue. *Cancer Res* **54**: 2615–2621

Katoh M, Hirai M, Sugimura T and Terada M (1996) Cloning, expression and chromosomal localization of Wnt-13, a novel member of the Wnt gene family. *Oncogene* **13**: 873–876

Korinek V, Barker N, Morin PJ, van Wichen D, de Weger R, Kinzler KW, Vogelstein B and Clevers H (1997) Constitutive transcriptional activation by a B-catenin-Tcf complex in APC–/– colon carcinomas. *Science* **275**: 1784–1787

LeJeune S, Huguet EL, Hamby A, Poulsom R and Harris AL (1995) Wnt 5a cloning, expression, and upregulation in human primary breast cancers. *Clin Cancer Res* **1**: 215–222

Miyoshi Y, Ando H, Nagase H, Nishisho I, Horii A, Miki Y, Mori T, Utsunomiya J, Baba S, Petersen G, Hamilton SR, Lomzer KW, Vogelstein B and Nakamura Y (1992) Germ-line mutations of the APC gene in 53 familial adenomatous polyposis patients. *Proc Natl Acad Sci USA* **89**: 4452–4456

Morin PJ, Sparks AB, Korinek V, Barker N, Clevers H, Vogelstein B and Kinzler KW (1997) Activation of B-catenin-Tcf signalling in colon cancer by mutations in β-catenin or APC. *Science* **275**: 1787–1790

Munemitsu SI, Albert B, Souza B, Rubinfield B and Polakis P (1995) Regulation of intracellular B-catenin levels by the adenomatous polyposis coli (APC) tumor-suppressor protein. *Proc Natl Acad Sci USA* **92**: 3046–3050

Nakagawa H, Murata Y, Koyama K, Fujiyama A, Miyoshi Y, Monden M, Akiyama T and Nakamura Y (1998) Identification of a brain-specific APC homologue, APCL, and its interaction with beta-catenin. *Cancer Res* **15**: 5176–5181

Nakamura T, Hamada F, Ishidate T, Anai K, Kawahara K, Toyoshima K and Akiyama T (1998) Axin, an inhibitor of the wnt signalling pathway, interacts with beta catenin, GSK-3beta and APC and reduces the beta catenin level. *Genes Cells* **3**: 395–403

Papkoff J, Rubinfeld B, Schryver B and Polakis P (1996) Wnt-1 regulates free pools of catenins and stabilizes APC-catenin complexes. *Mol Cell Biol* **16**: 2128–2134

Pennica D, Swanson TA, Welsh JW, Roy MA, Lawrence DA, Lee J, Brush J, Taneyhill LA, Deuel B, Lew M, Watanabe C, Cohen RL, Melhem MF, Finley GG, Qiurke P, Goddard AD, Hillan KJ, Gurney AL, Botstein D and Levine AJ (1999) Wisp genes are members of the connective-tissue growth-factor family that are upregulated in wnt-1-transformed cells and aberrantly expressed in human colon tumors. *Proc Natl Acad Sci USA* **95**: 14717–14722

Powell SM, Zilz N, Beazer-Barclay Y, Bryan TM, Hamilton SR, Thibodeau SN, Vogelstein B and Kinzler KW (1992) APC mutations occur early during colorectal tumorigenesis. *Nature (London)* **359**: 235–237

Pulford KA, Rigney EM, Micklem KJ, Jones M, Stross WP, Gatter KC and Mason DY (1989) Kp1: a new monoclonal antibody that detects a monocyte/macrophage associated antigen in routinely processed tissue sections. *J Clin Pathol* **42**: 414–421

Rocheleau CE, Downs WD, Lin R, Wittmann C, Bei Y, Cha YH, Ali M, Priess JR and Mello CC (1997) Wnt signaling and an APC-related gene specify endoderm in early *C. elegans* embryos. *Cell* **90**: 707–716

Rubinfield B, Souza B, Albert I, Munemitsu S and Polakis P (1995) The APC protein and E-cadherin form similar but independent complexes with a-catenin, B-catenin, and plakoglobin. *J Biol Chem* **270**: 5549–5555

Scott PAE, Smith K, Bicknell R and Harris AL (1997) A reliable external control for ribonuclease protection assays. *Nucleic Acids Res* **25** 1305–1306

Senior PV, Critchley D, Beck F, Walker RA and Varley JM (1988) The localisation of laminin mRNA and protein in the postimplantation embryo and placenta of mouse: an in situ hybridisation and immunohistochemical study. *Development* **104**: 431–446

Smith KJ, Johnson KA, Bryan TM, Hill DE, Markowitz S, Willson JKV, Paraskeve C, Petersen GM, Hamilton SR, Vogelstein B and Kinzler KW (1993) The APC gene product in normal and tumor cells. *Proc Natl Acad Sci USA* **90**: 2846–2850

Su LK, Vogelstein B and Kinzler KW (1993) Association of the APC tumor suppressor protein with catenins. *Science* **262**: 1734–1737

Takebayashi Y, Yamada K, Miyadera K, Sumizawa Y, Furukawa T, Kinoshita F, Aoki D, Okumura H, Yamada Y, Akiyama S and Aikou T (1997) The activity and expression of thymidine phosphorylase in human solid tumours. *Eur J Cancer* **32A**: 1227–1232

© 1999 Cancer Research Campaign 

van Es JH, Kirkpatrick C, van de Wetering M, Molenaar M, Miles A, Kuipers J, Destree O, Peifer M and Clevers H (1999) Identification of APC2, a homologue of the adenomatous polyposis coli tumour suppressor. *Curr Biol* **9**: 105–108

Vider BZ, Zimber A, Chastre E, Prevot S, Gespach C, Estlein D, Wolloch Y, Tronick SR, Gazit A and Yaniv A (1996) Evidence for the involvement of the Wnt 2 gene in human colorectal cancer. *Oncogene* **12**: 153–158

Zeng L, Fagotto F, Zhang T, Hsu W, Vasicek TJ, Perry WL 3rd, Lee JJ, Tilghman SM, Gumbiner BM and Constantini F (1997) The mouse fused locus encodes Axin, an inhibitor of the wnt signaling pathway that regulates embryonic axis formation. *Cell* **90**: 181–192

*© 1999 Cancer Research Campaign*
